# Hexatoma (Eriocera) guangxiensis sp. nov., a new crane fly in the *plumbicincta* species group (Diptera, Limoniidae) from China

**DOI:** 10.3897/zookeys.1289.196984

**Published:** 2026-08-14

**Authors:** Bing Zhang, Jun Yan, Yue Ning, Pin Liu, Minfei Yan, Qingming Liu, Ding Yang

**Affiliations:** 1 Department of Life Sciences, Collage of Chemistry and Chemical Engineering, Xianyang Normal University, Xianyang 712000, China Department of Life Sciences, Collage of Chemistry and Chemical Engineering, Xianyang Normal University Xianyang China; 2 Engineering Research Center of Loess Plateau Authentic Medicinal Herbs, Universities of Shaanxi Province, Xianyang 712000, China Department of Entomology, College of Plant Protection, China Agricultural University Beijing China; 3 The Youth Innovation Team of Shaanxi Universities, Xianyang 712000, China Engineering Research Center of Loess Plateau Authentic Medicinal Herbs, Universities of Shaanxi Province Xianyang China; 4 Yan’an Institute of Agricultural Sciences, Yan’an 716000, China The Youth Innovation Team of Shaanxi Universities Xianyang China; 5 Jiangxi Yalin Ecological Science Co., Ltd, Nanchang 330029, China Yan’an Institute of Agricultural Sciences Yan’an China; 6 Department of Entomology, College of Plant Protection, China Agricultural University, Beijing 100193, China Jiangxi Yalin Ecological Science Co., Ltd Nanchang China

**Keywords:** Crane flies, Limnophilinae, southern China, taxonomy

## Abstract

The Hexatoma (Eriocera) plumbicincta (Brunetti, 1911) species group is a distinctive and species-poor lineage within the subgenus Hexatoma (Eriocera) Macquart, 1838. Only two species of this species group had previously been recorded from China. In the present study, H. (E.) chrysomela (Edwards, 1921) is redescribed and newly recorded from Guizhou Province, Hunan Province, and Guangxi Zhuang Autonomous Region of China, and a new species of this species group, H. (E.) guangxiensis**sp. nov**., is described and illustrated from Guangxi Zhuang Autonomous Region and Yunnan Province. A key to the *plumbicincta* species group occurring in China is provided.

## Introduction

*Eriocera* Macquart, 1838 is a subgenus of the genus *Hexatoma* Latreille, 1809 in the family Limoniidae. It is distributed worldwide, with 567 known species and subspecies, of which 75 taxa are from the Palaearctic Realm, 34 taxa from the Nearctic Realm, 143 taxa from the Neotropical Realm, 30 taxa from the Afrotropical Realm, 290 taxa from the Oriental region, and five taxa from the Australasian/Oceania realms (Oosterbroek 2026). Thus, the subgenus is large and morphologically diverse, and it was confirmed to be non-monophyletic by Ribeiro (2008). It is characterised by the following characters: body medium-sized to large; antenna with 4–10 flagellomeres; wings often unpatterned through variously darkened, or with a conspicuous hyaline and yellow cross banded pattern, or abundantly spotted and dotted with brown; cell *dm* present; three or four branches of *M* reaching margin; clasper of gonostylus narrowed apically into a long, curved spine; lobe of gonostylus short, stout, and with setae; gonocoxite moderately stubby or elongate-cylindrical; interbase usually cylindrical, or triangular with a sharp spine at base, or with a two-layered membranous structure with spine at apex; aedeagus usually short and relatively inconspicuous or directed ventrally (Alexander and Lloyd 1914; Edwards 1921; Alexander 1948; Joseph 1977; Alexander and Byers 1981; Savchenko 1986; Podenas et al. 2006; Ribeiro 2008; Podeniene and Gelhaus 2015; Zhang et al. 2021; Podenas et al. 2022).

**Figure 1.**
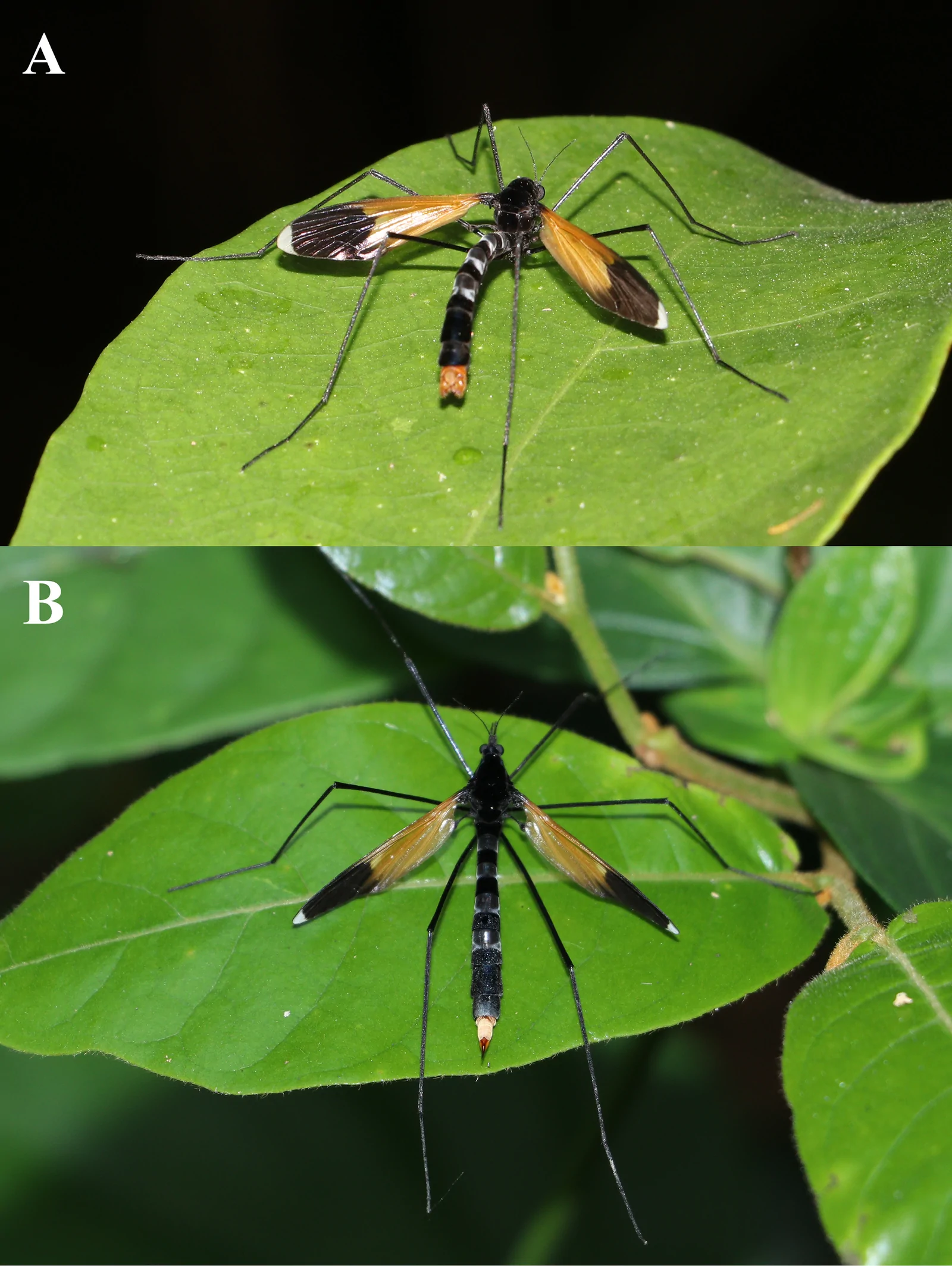
Habitus of Hexatoma (Eriocera) chrysomela. **A**. Male; **B**. Female.

**Figure 2.**
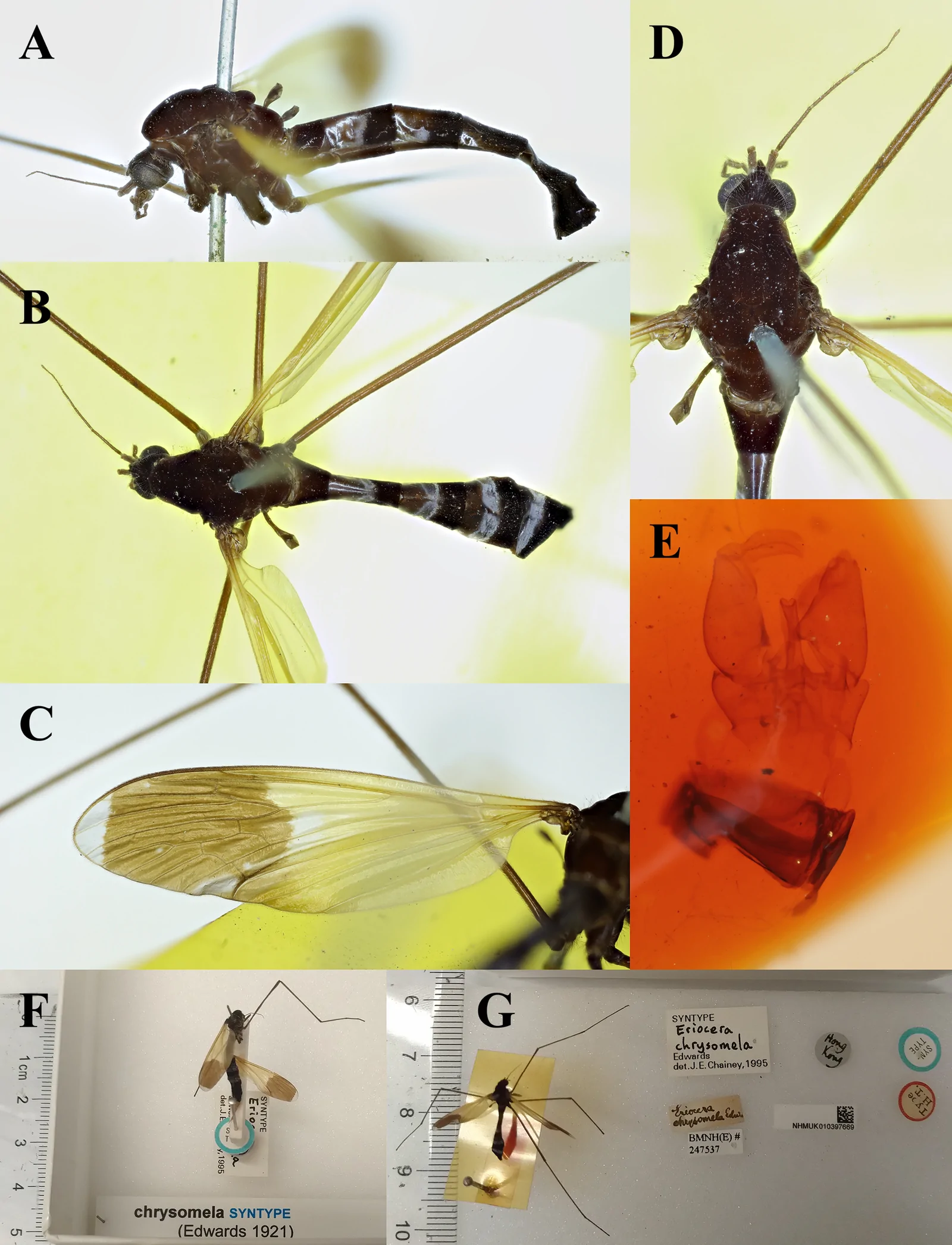
Hexatoma (Eriocera) chrysomela, type specimens, NHMUK. **A**. Male habitus, lateral view (holotype); **B**. Male habitus, dorsal view (holotype); **C**. Male wing (holotype); **D**. Male head and thorax, dorsal view (holotype); **E**. Terminalia, ventral view (holotype); **F**. Female habitus, dorsal view (syntype); **G**. Male habitus, dorsal view (holotype).

**Figure 3.**
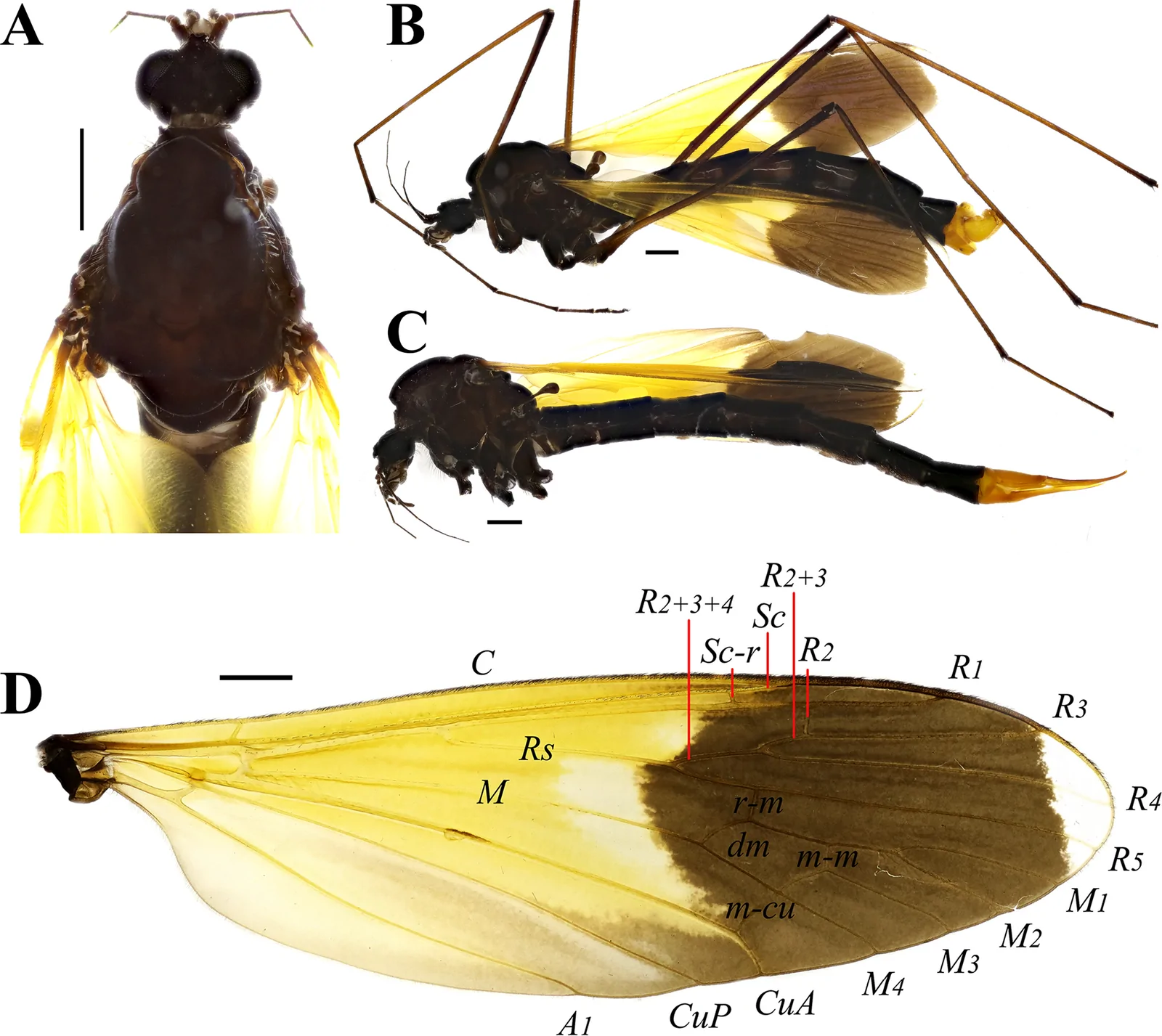
Hexatoma (Eriocera) chrysomela. **A**. Male head and thorax, dorsal view; **B**. Male habitus, lateral view; **C**. Female habitus, lateral view; **D**. Male wing. Scale bars: 1.0 mm.

**Figure 4.**
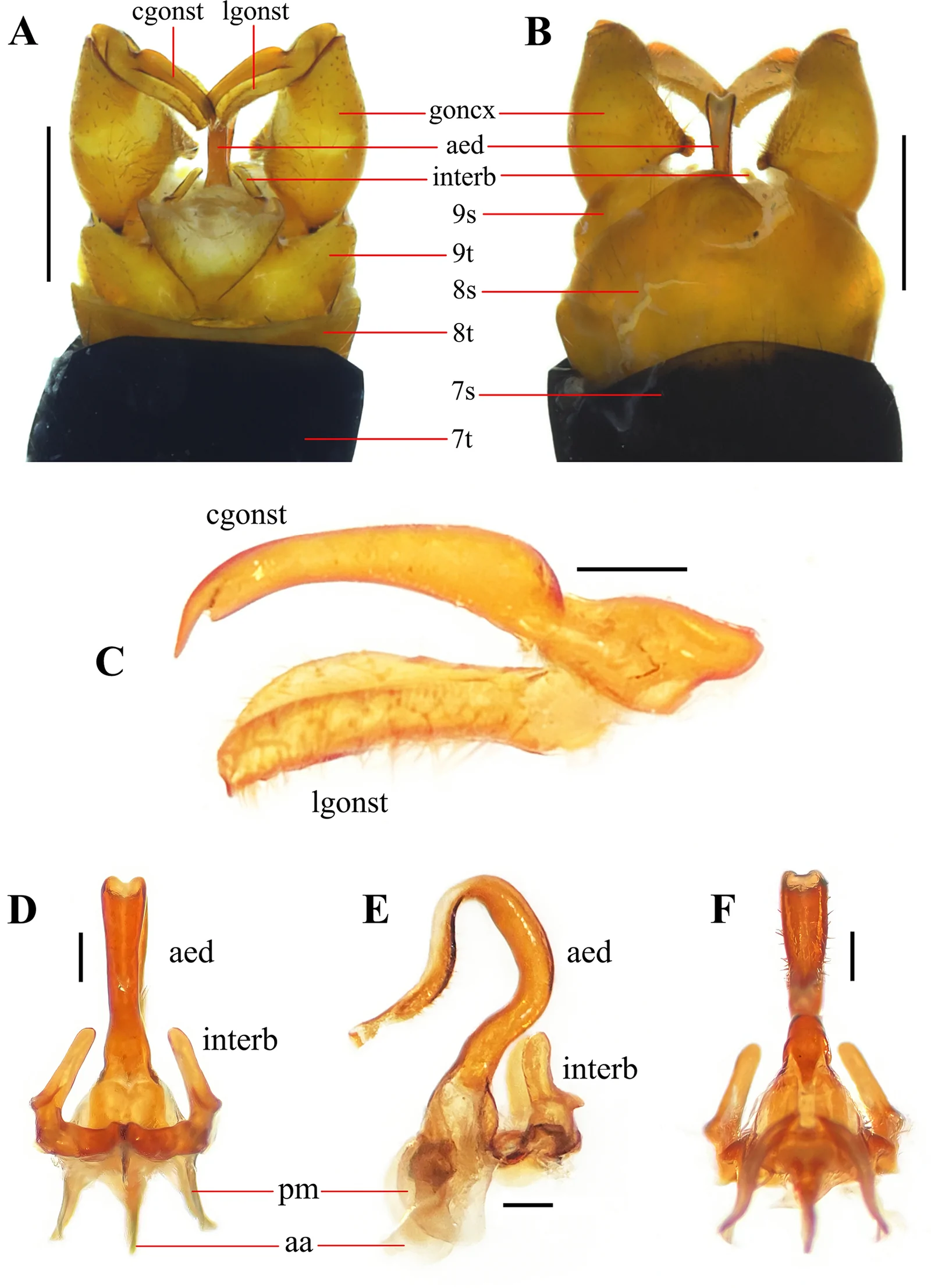
Male terminalia of Hexatoma (Eriocera) chrysomela. **A**. Terminalia, dorsal view; **B**. Terminalia, ventral view; **C**. Clasper of gonostylus and lobe of gonostylus, dorsal view; **D**. Aedeagal complex, dorsal view; **E**. Aedeagal complex, lateral view; **F**. Aedeagal complex, ventral view. Abbreviations: 7s = seventh sternite; 7t = seventh tergite; 8s = eighth sternite; 8t = eighth tergite; 9s = ninth sternite; 9t = ninth tergite; goncx = gonocoxite; cgonst = clasper of gonostylus; lgonst = lobe of gonostylus; interb = interbase; aed = aedeagus; pm = paramere; aa = aedeagal apodeme. Scale bars: 1 mm (**A, B**), 0.2 mm (**C–F**).

**Figure 5.**
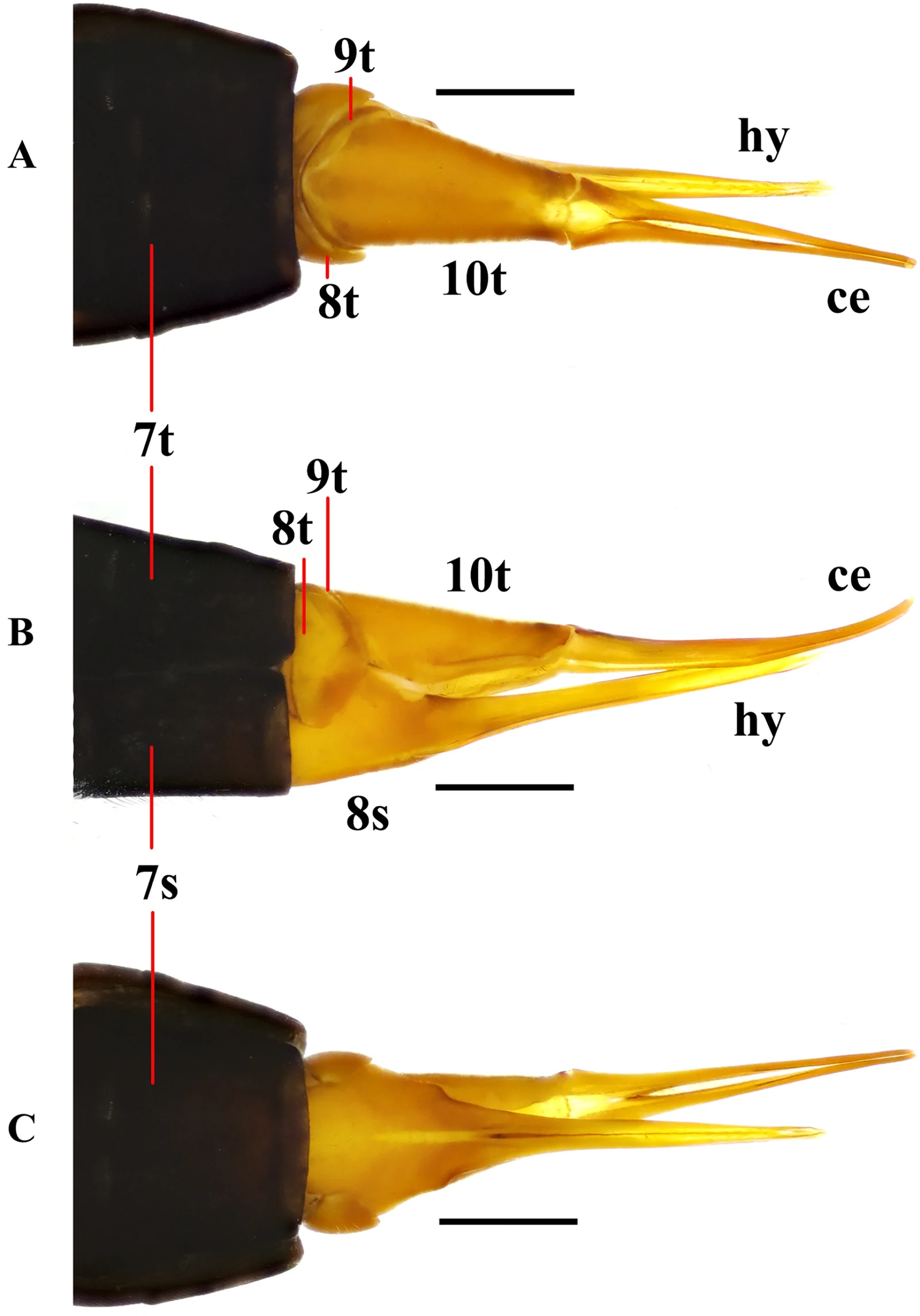
Female terminalia of Hexatoma (Eriocera) chrysomela. **A**. Ovipositor, dorsal view; **B**. Ovipositor, lateral view; **C**. Ovipositor, ventral view. Abbreviations: 7s = seventh sternite; 7t = seventh tergite; 8s = eighth sternite; 8t = eighth tergite; 9t = ninth tergite; 10t = tenth tergite; ce = cercus; hy = hypogynial valve. Scale bars: 1 mm.

The species group H. (E.) plumbicincta (Brunetti, 1911) possesses highly distinctive morphological characters within the subgenus *H.* (*Eriocera*). It is characterised by the following characters: head and thorax velvet-black; antenna 7-segmented (Fig. 2A–D); wing mainly dark brown, yellow, yellowish-brown, whitish-hyaline; with a whitish-hyaline, oblique medial stripe; a small whitish hyaline spot in cell *R_1_*; apex with a big whitish-hyaline stripearound *R_4_*; *M_1_* present, longer than its petiole; *m-cu* at outer end of cell *dm* (Figs 1A, 2C, 3D, 5B); male terminalia orangish yellow or reddish brown; ninth tergite short, with posterior margin concave; aedeagus extremely long, overall U-shaped, dorsal side robust, strongly sclerotised; ventral portion slender, rod-shaped, medially with numerous short setae; interbase rod-shaped, with a triangular process at base; paramere and aedeagal apodeme flattened in lateral view (Figs 1A, 2E, 4, 7) (Brunetti 1911; Edwards 1921; Alexander 1942; Joseph 1977; Men and Yu 2015). To date, only two species of the *plumbicincta* species group have been known to occur in China (Oosterbroek 2026): H. (E.) chrysomela (Edwards, 1921) from Fujian, Guangdong, Hong Kong and Jiangxi Province and H. (E.) sincera Alexander, 1942 from Yunnan Province. Currently, H. (E.) chrysomela is reported for the first time from Guangxi, Guizhou, and Hunan Province, one new species H. (E.) guangxiensis sp. nov. is described and illustrated from Guangxi and Yunnan Province. A key to the species group H. (E.) plumbicincta from China is provided.

## Materials and methods

The specimens were examined and illustrated using a ZEISS Stemi 2000-c stereomicroscope. Details of their colouration were checked in specimens immersed in 75% ethyl alcohol. Male genitalia were prepared by macerating the apical portion of the abdomen in cold 10% NaOH for 12–15 h. After examination, the genitalia were transferred to fresh glycerol (C_3_H_3_O_3_) and stored in a microvial pinned beneath the respective specimen. The studied specimens are deposited in the Entomological Museum of China Agricultural University (**CAU**), Beijing, China. Some type and non-type material H. (E.) chrysomela (Edwards, 1921) used in this paper were borrowed from the Natural History Museum, London, UK (**NHMUK**).

The terminology used for the wing veins follows the interpretation of Cumming and Wood (2017) and Jong (2017). The terminology for the male terminalia follows that of Ribeiro (2006, 2008).

## Taxonomy

### Species list of Hexatoma (Eriocera) plumbicincta group recorded in China

Hexatoma (Eriocera) chrysomela (Edwards, 1921).

Hexatoma (Eriocera) guangxiensis sp. nov.

Hexatoma (Eriocera) sincera Alexander, 1942.

### Key to species of the group Hexatoma (Eriocera) plumbicincta from China

**Table:** 

1	Wing with cells *Rs* and *M* extensively dark brown; a whitish-hyaline, oblique, medial stripe, starting from vein *R* and ending at vein *CuA* (Alexander 1942: 183)	**Hexatoma (Eriocera) sincera**
–	Wing with cells *Rs* and *M* extensively yellow; a whitish-hyaline, oblique, medial stripe, starting from vein *Rs* and ending at vein CuA (Figs 1A, 2C, 3D, 6B)	**2**
2	Femur brownish black (Figs 1A, 2A–D, 3B); cells *CuP* and *A_1_* paler yellow basally, becoming darker distally (Figs 1A, 2C, 3D); male terminalia orangish yellow; ninth tergite with a deep, triangular incision at concavity; interbase rod-shaped, uniform in width, slightly curved dorsad at apex; paramere fan-shaped in lateral view; aedeagal apodeme laterally fan-shaped (Fig. 4)	**Hexatoma (Eriocera) chrysomela**
–	Femur yellow at basal third, with posterior margin more blackish brown (Fig. 6A); basal third of cells *CuA*, *CuP*, and *A_1_* more greyish yellow, approaching dark brown distally (Fig. 6B); male terminalia reddish brown; ninth tergite with a U-shaped concavity; interbase relatively straight, elongate-conical, basal part thick, gradually tapering toward apex; paramere subpentagonal in lateral view; aedeagal apodeme subtriangular in lateral view (Fig. 7)	**Hexatoma (Eriocera) guangxiensis sp. nov**.

#### Hexatoma (Eriocera) chrysomela

Taxon classificationAnimaliaDipteraLimoniidae

(Edwards, 1921)

09F2D7B3-BF4D-5310-B9D2-88DE6AF5E1DC

Figs 1, 2, 3, 4, 5

Eriocera chrysomela Edwards, 1921: 88.
Hexatoma (Eriocera) chrysomela —Alexander, 1937: 389; Alexander and Alexander 1973: 143; Men and Yu 2015: 164; Li et al. 2023: 774.

##### Type material examined.

China • holotype ♂; Hong Kong; 1861; J.C. Bowring leg.; BMNH(E) # 247537, NHMUK010397669 • syntype 1♀; Hong Kong; 1995; det. J.E. Chainey leg.; NHMUK.

##### Additional material examined.

China • 3♂♂, 2♀♀; Fujian, Jiangle; 10 October 1985; Liqin Zhang leg.; CAU Limn: 129167**–**129171; genitalia dissected • 1♂; Guizhou, Shiqian, Jinxing Village; 21 July 1988; Yijun Wang leg.; CAU: Limn: 129172 • 1♂; Fujian, Fuzhou, Cangshan District, Fujian Agriculture and Forestry University; 12 October 1985; Mingbing Lu leg.; CAU Limn: 129173; genitalia dissected • 1♀; Guangdong, Shixing, Chebaling; 10–12 July 2003; Xingyue Liu leg.; CAU Limn: 129174; genitalia dissected • 1♂ (in alcohol); Guangdong, Zengcheng, Nankunshan National Forest Park; 14–15 July 2003; Xingyue Liu leg.; CAU Limn: 129175 • 1♂ (in alcohol); Guangdong, Shaoguan; 18 July 2003; Xingyue Liu leg.; CAU Limn: 129176 • 1♂ (in alcohol); Guangdong, Longmen, Nankunshan National Forest Park; 1 April 2005; Bo Wang leg.; CAU Limn: 129177 • 1♀ (in alcohol); Guangxi, Huaping, Cujiangzhan; 21 August 2020; Jiuzhou Liu leg.; CAU Limn: 129178 • 1♀ (in alcohol); Hunan, Chengbu, Baimaoping Town, Houjiazhai; 8 September 2020; Qicheng Yang leg.; CAU Limn: 129179 • 1♂1♀ (in alcohol); Guangdong, Shixing, Chebaling; 20 October 2020; Ding Yang & Hui Dong leg.; CAU Limn: 129180**–**129181; genitalia dissected • 1♂ (in alcohol); Guangxi, Fuchuan, Xiayuandong Village; 14 August 2021; Xiaodong Cai leg.; CAU Limn: 129182; genitalia dissected.

##### Diagnosis.

Head and thorax velvet-black; femur brownish black; wing with yellow area extending from base beyond mid-length of wing; cells *CuP* and *A_1_* basally pale yellow, distally becoming darker; male terminalia orangish yellow; ninth tergite with posterior margin strongly concave, with a deep triangular incision at concavity, width approximately half that of terminalia; aedeagus extremely long, ventrally bent over and almost U-shaped, somewhat flattened at middle, strongly sclerotised at base (dorsally), distally more slender, with numerous short setae on distal (ventral) section, tip slender; interbase rod-shaped, uniform in width, with a triangular process at base, slightly curved dorsad at apex and apex obtuse, rounded; paramere flattened, fan-shaped in lateral view; aedeagal apodeme flattened, laterally fan-shaped, about ½ size of paramere.

##### Redescription.

**Male**. Body length 17.2–19.6 mm, wing length 14.8–15.8 mm, antenna length 3.2–3.4 mm (*n* = 11).

***Head*** (Figs 1A, 2A, 2B, 2D, 3A, 3B) velvet-black with blackish-grey setae. Antenna 7-segmented, brownish black, scape cylindrical, pedicel short, flagellomeres cylindrical with short verticils; rostrum and palpus brownish black with black setae; vertex tubercle small with a forward-facing tip.

***Thorax*** (Figs 1A, 2A, 2B, 2D, 3A, 3B) velvet-black with blackish grey setae. Legs: coxa and trochanter velvet-black, with dense, blackish-grey setae; femur brownish black, with posterior margin become blacker and thicker; tibia and tarsus black, with brownish-black setae. Wing with surface multicoloured (Figs 1A, 2C, 3D), with a yellow area extending from base beyond mid-length of wing; cells *CuP* and *A_1_* pale yellow basally, becoming darker distally; whitish hyaline, oblique medial stripe, starting from vein *Rs* and ending at vein *CuA*; small, indistinct, pale-yellow spot in cell *R_1_*; tip of wing with a large, whitish-hyaline stripe around *R_4_*, originating at *R_3_* and terminating at *M_1_*, length of spot equal to that of *M_1_*. Venation: *R_2+3_* a little longer than *R_2_*; *R_2+3+4_* approximately 2 times as long as *R_2_*; *M_1_* approximately 2 times as long as *M_1+2_*; *m-cu* at outer end of cell *dm*. Halter velvet-blackish brown.

***Abdomen*** (Figs 1A, 2A, 2B, 3B) with short, blackish-grey setae. First segment velvet-black; segments 2–5 elongate, base shining black, middle with pruinosity, tip velvet-black; segments 6–7 velvet-black, relatively short; segment 8 orangish yellow, very short.

***Male terminalia*** (Figs 1A, 2E, 4) orangish yellow, with dense brownish setae. Ninth tergite short, posterior margin strongly concave, with a deep, triangular incision at concavity, width approximately half that of terminalia (Fig. 4A). Gonocoxite nearly triangular, basally broad, tip narrowed, and with dense setae; inner side of gonocoxite with darkened, triangular extension (Fig. 4A, B). Clasper of gonostylus slender, tapering to acute apex, with terminal spine decurved (Fig. 4C). Lobe of gonostylus short, stout, and with dense setae along ventral margin; apex rounded. Aedeagus extremely long, bent over ventrally and almost U-shaped, somewhat flattened at middle, elongated at base (dorsally), more slender distally, with numerous short setae on distal (ventral) section, tip slender (Fig. 4D–F). Interbase rod-shaped, uniform in width, with a triangular process at base, slightly curved dorsad at apex, apex obtuse and rounded. Paramere flattened, fan-shaped in lateral view. Aedeagal apodeme flattened, fan-shaped laterally, about ½ size of paramere (Fig. 4D–F).

**Female**. Body length 19.3**–**25.4 mm, wing length 15.8**–**18.6 mm, antenna length 3.1–3.4 mm (*n* = 7). Female generally resembling male by body colouration (Figs 1B, 2F, 3C).

***Female terminalia*** (Fig. 5). Terminal segments from the eighth posteriorly orangish yellow, with brownish setae, nearly 1/4 as long as abdomen. Cercus orange, 1.5 times as long as tenth tergite, upcurved on distal part. Hypogynial valve orange, distal part yellow; hypogynial valve more than twice as long as eighth sternite, almost straight, tip ending at near level of 2/3 of cercus.

##### Distribution.

China (Fig. 8): Fujian (Fuzhou; Jiangle; Liung-chon-san), Guangdong (Longmen; Shixing), Guangxi (Fuchuan; Huaping), Guizhou (Shiqian), Hong Kong, Hunan (Chengbu), Jiangxi (Hong San).

##### Remarks.

This species is reported for the first time from Guangxi, Guizhou, and Hunan provinces.

#### Hexatoma (Eriocera) guangxiensis

Taxon classificationAnimaliaDipteraLimoniidae

Zhang & Yang sp. nov.

3812FFC5-3E3F-5960-B05B-A8064016E7F8

https://zoobank.org/9A572851-F87C-4FFD-B776-8840BDAA20D2

Figs 6, 7

##### Type material.

***Holotype***: China • ♂; Guangxi, Pingxiang; 11 May 1963; Jikun Yang leg.; CAU Limn: 129183; genitalia dissected.

***Paratypes***: China • 1♂; Guangxi, Pingxiang; 11 May 1963; Jikun Yang leg.; CAU Limn: 129184; genitalia dissected • 1♂; Guangxi, Pingxiang, Xiashi Town; 7 May 1963; Jikun Yang leg.; CAU Limn: 129185 • 1♂; Yunnan, Hekou, Nanxi Town; 17 May 2006; Jianxin Cui and Junhua Zhang leg.; CAU Limn: 129186; genitalia dissected.

##### Diagnosis.

Head velvet-black; thorax velvet-blackish brown; femur yellow on basal third and posterior margin more blackish brown and thicker; wing with yellow area extending from base beyond mid-length of wing; basal third of cells *CuA*, *CuP* and *A_1_* more greyish yellow, approaching dark brown distally; male terminalia reddish brown; aedeagus extremely long, bent over ventrally almost U-shaped, somewhat thicker at middle, strongly sclerotised at base (dorsally), distally more slender and with numerous short setae on distal (ventral) section; interbase relatively straight, elongate-conical, basal part thick, gradually tapering toward apex, with a triangular process at base, apex obtuse and rounded; paramere flattened, subpentagonal in lateral view; aedeagal apodeme flattened, subtriangular in lateral view.

##### Description.

**Male**. Body length 18.2**–**25.3 mm, wing length 13.3**–**18.5 mm, antenna length 3.2–3.8 mm (*n* = 4).

**Figure 6.**
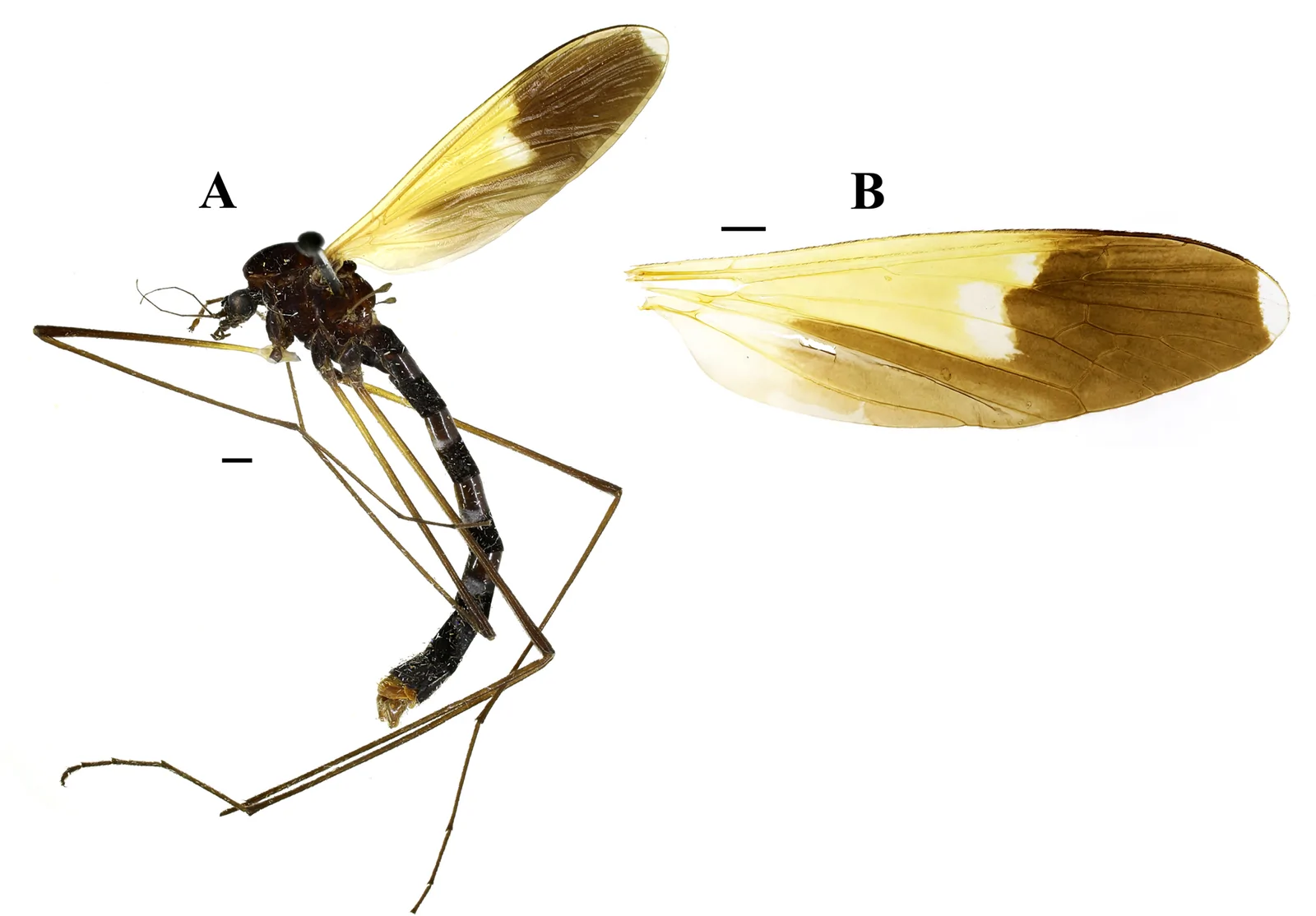
Hexatoma (Eriocera) guangxiensis sp. nov. **A**. Male holotype, lateral view; **B**. Wing. Scale bars: 1 mm.

***Head*** (Fig. 6A) velvet-black with blackish-grey setae. Antenna 7-segmented, blackish brown; scape cylindrical; pedicel short; flagellomeres cylindrical, with short verticils; rostrum and palpus blackish brown, with black setae; vertex tubercle small, with a forward-facing tip.

***Thorax*** (Fig. 6A) velvet-blackish brown, with blackish-grey setae. Legs: coxa velvet-blackish brown; trochanter dark brown, with dense, blackish-grey setae; femur yellow on basal 1/3, posterior margin becoming more blackish brown and thicker; tibia and tarsus dark, with brownish-black setae. Wing with surface multicoloured (Fig. 6B); yellow area extending from base beyond mid-length of wing; basal third of cells *CuA*, *CuP*, and *A_1_* more greyish yellow, approaching dark brown distally; a whitish-hyaline, oblique stripe medially, starting from vein *Rs* and ending at vein *CuA*; small, whitish-hyaline spot in cell *R_1_*, originating at *R* and terminating at *R_5_*; tip of wing with a big, whitish-hyaline stripe around *R_4_*, originating at *R_3_* and terminating at *R_5_*, length of spot equal to that of *M_2_*. Venation: *R_2+3_* as long as *R_2_*; *R_2+3+4_* approximately 1.5 times as long as *R_2+3_*; *M_1_* approximately 2 times as long as *M_1+2_*; *m-cu* at outer end of cell *dm*. Halter velvet-brownish grey.

***Abdomen*** (Fig. 6A) with short, blackish-grey setae. First segment velvet-black; segments 2–5 elongate, base shining black, middle with pruinosity, tip velvet-black; segments 6–7 velvet-black, relatively short; segment 8 reddish brown, very short.

**Figure 7.**
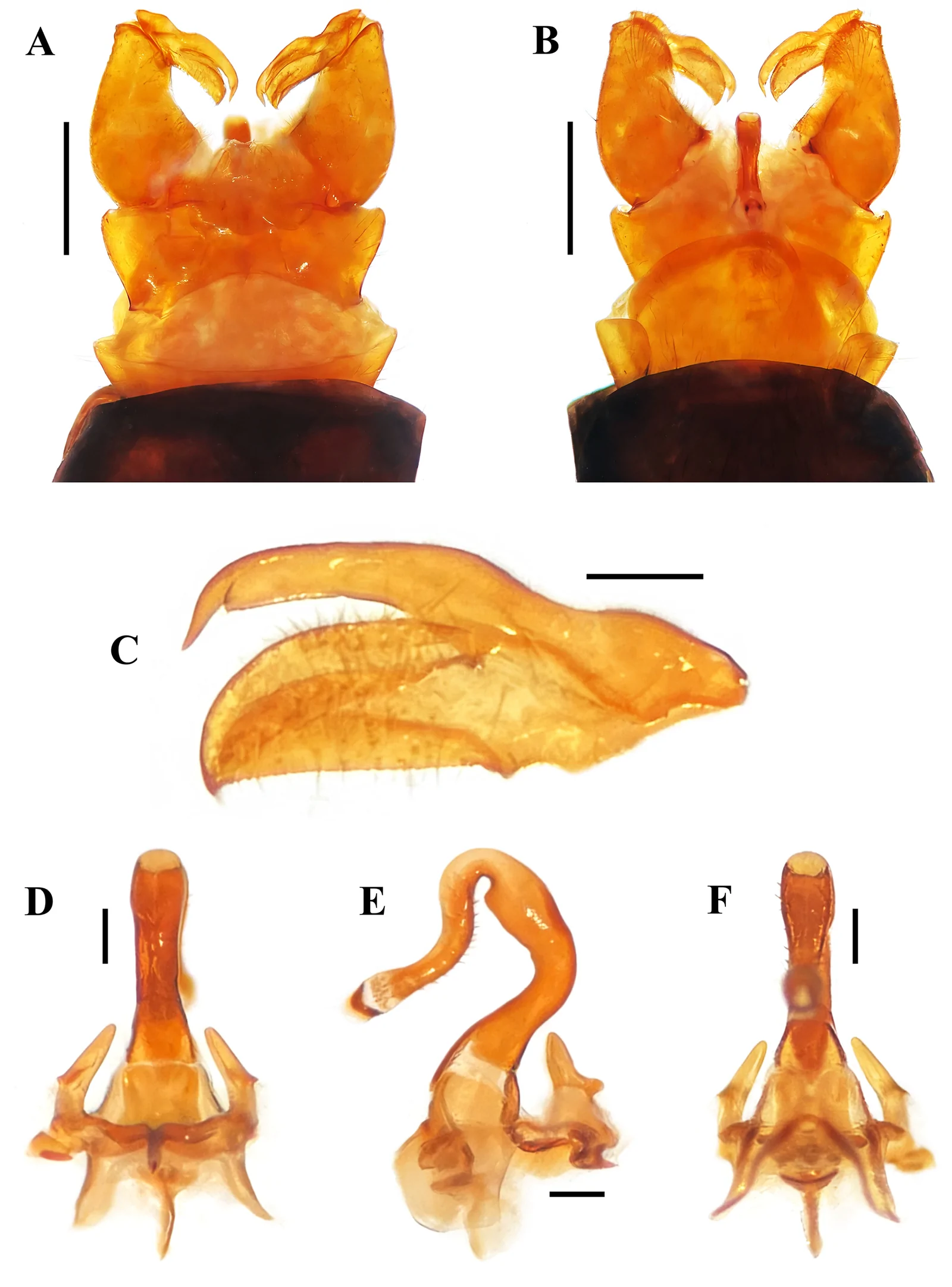
Male terminalia of Hexatoma (Eriocera) guangxiensis sp. nov. **A**. Terminalia, dorsal view; **B**. Terminalia, ventral view; **C**. Clasper of gonostylus and lobe of gonostylus, dorsal view; **D**. Aedeagal complex, dorsal view; **E**. Aedeagal complex, lateral view; **F**. Aedeagal complex, ventral view. Scale bars: 1 mm (**A, B**), 0.2 mm (**C–F**).

***Male terminalia*** (Fig. 7) reddish brown, with dense, brownish setae. Ninth tergite short, posterior margin with strong, U-shaped concavity, width approximately 1/3 of terminalia (Fig. 7A). Gonocoxite triangular; base of gonocoxite wider; inner side of gonocoxite with triangular extension; tip with dense, stout setae; end of gonocoxite relatively narrow (Fig. 7A, B). Clasper of gonostylus slender, tapering to acute apex; terminal spine decurved (Fig. 7C). Lobe of gonostylus short and stout, with dense setae along margin; apex rounded (Fig. 7C). Aedeagus extremely long, bent over ventrally almost U-shaped, somewhat thicker in the middle, strongly sclerotised at base (dorsally), distally more slender, with numerous short setae on distal (ventral) section; tip thicker (Fig. 7D–F). Interbase relatively straight, elongate-conical, basal part thick, gradually tapering toward apex, with a triangular process at base, apex obtuse and rounded. Paramere flattened, subpentagonal in lateral view. Aedeagal apodeme flattened, subtriangular in lateral view, approximately 1/3 the size of paramere (Fig. 7D–F).

**Female**. Unknown.

##### Distribution.

**Figure 8.**
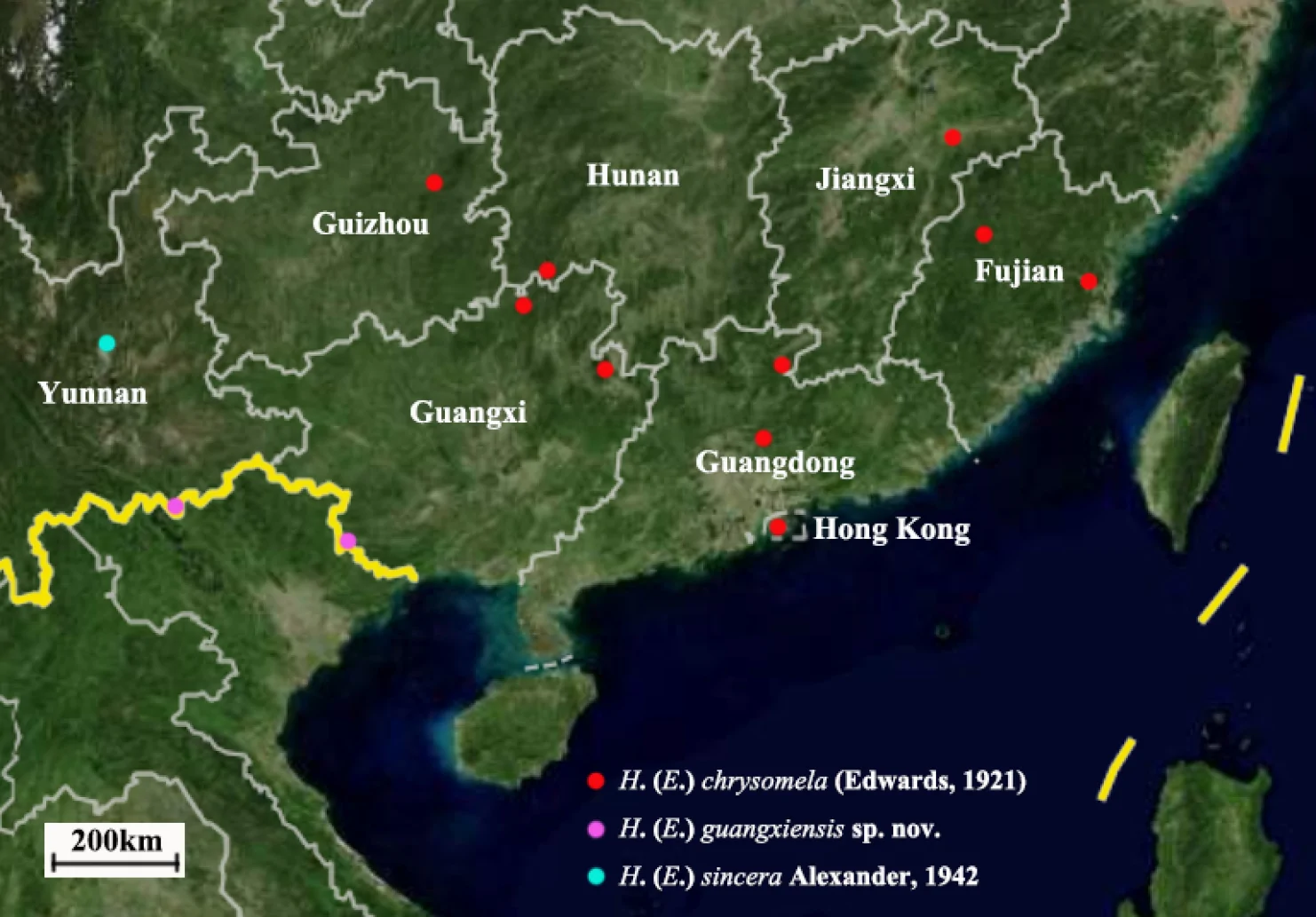
Worldwide distribution of Hexatoma (Eriocera) chrysomela, H. (E.) guangxiensis sp. nov., and H. (E.) sincera.

China (Fig. 8): Guangxi (Pingxiang), Yunnan (Hekou).

##### Etymology.

This species is named after Guangxi Zhuang Autonomous Region, where its type locality is situated.

##### Remarks.

The new species is very similar to H. (E.) chrysomela in having similar body colouration and wing venation, but it can be separated by the following: femur yellow on basal third andposterior margin more blackish brown; basal third of cells *CuA*, *CuP*, and *A_1_* more greyish yellow, approaching dark brown distally (Fig. 6). In H. (E.) chrysomela, the femur is brownish black; cells *CuP* and *A_1_* are basally pale yellow but darker distally (Figs 1A, 2A–D, 3B, 3D). Both species can be also separated by the features of male genitalia. In H. (E.) guangxiensis sp. nov., male terminalia are reddish brown, the ninth tergite has a U-shaped concavity; the interbase is relatively straight, elongate-conical, with the basal part thick, and gradually tapering toward apex; the paramere is subpentagonal in lateral view; the aedeagal apodeme is subtriangular in lateral view, approximately 1/3 of the size of the paramere (Fig. 7). In H. (E.) chrysomela, male terminalia are orangish yellow; the ninth tergite has a deep, triangular incision at a concavity; the interbase is rod-shaped, uniform in width, and slightly curved dorsad at the apex; the paramere is fan-shaped in lateral view; and the aedeagal apodeme is fan-shaped laterally, about ½ of the size of the paramere (Fig. 4).

#### Hexatoma (Eriocera) sincera

Taxon classificationAnimaliaDipteraLimoniidae

Alexander, 1942

D4432A52-163F-5A1A-ADE4-A5E80AB0BC49

Hexatoma (Eriocera) sincera Alexander, 1942: 182.
Hexatoma (Eriocera) sincera : Alexander & Alexander, 1973: 153; Men and Yu 2015: 164.

##### Diagnosis.

Body length 14 mm, wing length 12.5 mm, antenna length 3.5 mm (Alexander 1942: 182). Head velvet-black; thorax uniformly opaque velvet-black; femur dark brown; wing with dark-brown ground colour; base of wing 1/6 yellow; cells *C* and *Sc* yellow; wing with a whitish-hyaline, oblique stripe medially, starting from vein *R* and ending at vein *CuA*; apex with a small, whitish-hyaline stripe around *R_4_*, length of spot equal to that of *m-cu*; *R_2+3_* a little longer than *R_2_*; *R_2+3+4_* a little longer than *R_2+3_*; *M_1_* approximately 1.5 times as long as *M_1+2_*; *m-cu* at outer end of cell *dm* (Alexander 1942: 178, fig. 4); abdomen opaque black; segments 2–5, inclusive, with heavily light-grey-pruinose basal rings, especially heavy on distal portion; pruinose area including not more than ½ total length of segment; sternites with basal rings more plumbeous and glabrous rather than pruinose; hypopygium and preceding segment orange (Alexander 1942: 183).

##### Distribution.

China (Fig. 8): Yunnan (Yunnan-fu).

## Supplementary Material

XML Treatment for Hexatoma (Eriocera) chrysomela

XML Treatment for Hexatoma (Eriocera) guangxiensis

XML Treatment for Hexatoma (Eriocera) sincera

## Additional information

### Artificial Intelligence (AI) use

The authors accept full responsibility for the content of the manuscript, including the disclosure of any use of AI.

No AI tools were used in the preparation of this manuscript.

### Author ORCIDs

B. Zhang https://orcid.org/0000-0002-3996-1760

D. Yang https://orcid.org/0000-0002-7685-3478

### Data availability

All of the data that support the findings of this study are available in the main text.
